# Supplementary material for: Examining SEB skills’ incremental validity over personality traits in predicting academic achievement
Source: PLoS One. 2024 Jan 3;19(1):e0296484. doi: 10.1371/journal.pone.0296484 (PMC10763938; doi:10.1371/journal.pone.0296484)
Supplement: Attachment — Submitted filename: Recommendation.docx [file pone.0296484.s002.docx]

The present research, authors tested whether particular SEB skills predicted high school students’ ACT scores beyond their Big Five personality traits. Significant changes in the incremental regression coefficients were used as comparison parameters. The results enrich the predictors of academic achievement, and have certain reference significance for further guiding students' learning from the perspective of SEB, and test the validity of the SEB measurement questionnaire.

Therefore, I suggest accepting this manuscript.

Please revise the following aspects：

1. Supplement illustrate the theoretical and measured differences between SEB and Big Five personality traits and the need to predict academic achievement with SEB.
2. Soto, C. J., Napolitano, C., Sewell, M., Yoon, H. R., & Roberts, B. (2021). An Integrative Framework for Conceptualizing and Assessing Social, Emotional, and Behavioral Skills: The BESSI.

Soto, C. J. , Napolitano, C. M. , Sewell, M. N. , Yoon, H. J. , & Roberts, B. W. . (2022). An integrative framework for conceptualizing and assessing social, emotional, and behavioral skills: the bessi. *Journal of personality and social psychology,* *123*(1), 192-222.
